# Familiar Music as an Enhancer of Self-Consciousness in Patients with Alzheimer's Disease

**DOI:** 10.1155/2013/752965

**Published:** 2013-09-11

**Authors:** Eva M. Arroyo-Anlló, Juan Poveda Díaz, Roger Gil

**Affiliations:** ^1^Department of Psychobiology and Methodology of Behaviour Sciences, Neuroscience Institute of Castilla-León, University of Salamanca, 37007 Salamanca, Spain; ^2^Memory Clinic, 37002 Salamanca, Spain; ^3^Department of Neurology, University Hospital, CHU La Milétrie, 86000 Poitiers, France

## Abstract

The main objective of this paper is to examine the impact of familiar music on self-consciousness (SC) in patients with Alzheimer's disease (AD). For this purpose, two AD groups of 20 patients matched by age, educational level, gender, illness duration, and cognitive state were assessed using an SC questionnaire before and after music intervention. The SC questionnaire measured several aspects: personal identity, anosognosia, affective state, body representation, prospective memory, introspection and moral judgments. One AD group received familiar music stimulation and another AD group unfamiliar music stimulation over three months. The AD patients who received a familiar music intervention showed a stabilization or improvement in aspects of SC. By contrast, control AD group showed a deterioration of most of the SC aspects after unfamiliar music stimulation, except the SC aspects of body representation and affective state. Familiar music stimulation could be considered as an enhancer of SC in patients with Alzheimer's disease.

## 1. Introduction

Alzheimer's disease (AD) is a degenerative dementia characterized by a progressive decline in cognitive function. In the absence of effective treatment for the causes of AD, nonpharmacological interventions have prompted increasing scientific interest in cognition and/or behavioural techniques for individuals with dementia. Nonpharmacological interventions may vary in terms of the degree to which the program is individualised, the content of the activity, and the nature of the facilitation. A broad array of such interventions has been developed over the past two decades, such as cognitive training [1], sensory stimulation [2], music therapy [3], and motor stimulation [4] (see Trinh et al., 2003 [5] for a review).

Among those interventions, music therapy seems to be relatively effective and the least harmful one. Several works have demonstrated that music memory is relatively preserved in patients with moderate to severe AD in spite of, otherwise, severe overall impairment [6–9]. However, some reports have described impaired music memory in AD [10–12]. Baird and Samson [13] suggested that perhaps procedural memory for musical stimuli remain intact but not musical episodic memory. Furthermore, several studies [14–17] showed that semantic memory for melody may be preserved in AD.

Pharmacological interventions are available but have limited ability to treat many of the AD symptoms. Music therapy has the ability to alleviate some symptoms of dementia and permits remarkable responses to be elicited in patients [18]. Vink et al. [19] reviewed ten studies which assessed whether music therapy can diminish symptoms of dementia. According to these studies, moderate effects of any form of music therapy may be effective in reducing dementia troubles. Thus, those studies have showed the benefits of music therapy in Alzheimer's patients, focusing either on the improvement of healthy cognition, such as autobiographical memory [20–22], semantic memory [23], language ability [24], and cognitive function [21], or on neuropsychiatric symptoms, such as agitation [25–27], apathy [28], depression, and anxiety [29]. Furthermore, several studies showed that the musical familiarity can elicit certain affective responses [20, 24, 30–33], even also new musical stimuli could do it [34]. But despite ten studies describing positive effects of music therapy, Vanstone et al. [15] found some methodological limitations which impeded valid results and inferences.

 To our knowledge no work has directly examined effects of music on self-consciousness (SC) and never in patients with AD. SC is thought to be unique to humans and certain great apes [35]. SC is the subject's ability to understand his/her own states of consciousness. The subject can thus separate himself from his perception and realize that he is in the process of perceiving. SC or reflexive consciousness allows the subject to be the object of his awareness. At the same time, because he knows that he is thinking, he is the object of his awareness. The aphorism of R. Descartes (“I think, therefore I am”) shows that subject can realize that he is thinking and also deduce from that the reality of his existence. Self-consciousness includes the consciousness of one's own mental states, such as perceptions, attitudes, opinions, and intentions to act. Representing and integrating such mental states into a common framework, which represents the integrity of our own mind, requires the ability to take a self- or first-person perspective among other constitutive features, such as experiences of agency or transtemporal unity [36]. Thus, SC is multifaceted [37]. Self-consciousness is consciousness of the body, that is, of its morphological characteristics, as well as its position and mobilization in space (i.e., we are well aware of being brown or blond, big or small, etc.). It is also the consciousness of perception, which can disrupt sectorally, for example, in cortical blindness or even unilateral spatial agnosia. Thus, when we see, hear, smell, feel, or anything else that we do we know that we see, hear, smell, feel,…. But beyond aspects of the consciousness of the body and perception, it is the consciousness of one's own autobiography [38]; it is inseparable from memory, thanks to the identity which each human being constructs for him or herself (“It is the same self now it was then”). Additionally, SC is the consciousness of one's own projects [39] and so it is, at once, an instance of anticipation, judgement, and decision. Finally, SC is a moral consciousness that allows human beings to make judgments about their thoughts and actions; it also allows him to think and act in the world he lives in accordance with the choice posed by moral reflection. In the extreme sense, the impairment of self-consciousness involves the subject no longer being conscious of existing. Throughout life, SC assembles a stream of perceptions, thoughts, recollections or memories, projects and actions which express the permanence and coherence of the ego and thus of the human person. Conway and Pleydell-Pearce [40] proposed that autobiographical memory is constructed within a self-memory system (SMS), a conceptual model composed of an autobiographical knowledge base and the working self. That autobiographical knowledge base contains knowledge of the self, used to provide information on what the self is, what the self was, and what the self can be, in order to make up the overall life story of an individual [41].

SC can be the object of scientific study, searching for impairment of SC in a patient requires considering which aspects of SC are altered or preserved. Thus, Gil et al. [39] elaborated a self-consciousness questionnaire (SCQ) which assesses seven aspects of SC: personal identity, anosognosia, affective state, body representation, future memory, introspection and moral judgments. SCQ is composed by fourteen questions (see the questionnaire), in order to obtain information from the patient about those seven aspects of SC (i.e., “What is your name (surname and first name)?”, concerning personal identity aspect).

The self-consciousness questionnaire containing 14 questions selected in order to evaluate some aspects of SC [39] is as follows.

1. What is your name (surname and first name)?
2. Why have you come to see me?
3. Do you have any health problems that prevent you from leading a normal life?
4. Do you have any problems with your memory?
5. Have you had a job? What was it?
6. What is the first name of your spouse (or partner)?
7. What is your mother's first name?
8. Do you feel happy or unhappy? Why?
9. Would you say that you are rather fair or dark haired?
10. Are you now sitting, standing, or lying down?
11. What are you planning to do shortly or tomorrow?
12. If you had to live your life over again, is there anything you would like to change? What?
13. Is it a good thing or a bad thing to tell a lie? Why?
14. Is it a good thing or a bad thing to give some money or some food to someone who is starving? Why?

Alzheimer's disease causes impairment of memory, in particular episodic and autobiographical memories, and difficulties in interaction between the self and the world, because AD is also characterized by disturbances of language, praxis, gnosis, and so forth. Gil et al. [39] studied SC in forty-five AD patients through the self-consciousness questionnaire. The authors of that work found that several aspects of SC were not impaired to the same degree. The most disturbed ones were awareness of cognitive deficiencies, moral judgements and prospective memory. The least disturbed aspects were awareness of identity and of mental representation of the body. 

 Since music therapy might be able to enhance aspects such as the affective state, behaviour, life experiences or a person's life history by providing access to lost memories of life events and people [20, 21, 24, 25]—which are considered aspects of SC—, we set out to determine whether familiar songs could enhance self-consciousness in patients with Alzheimer's disease. So, the purpose of our study was to examine the impact of familiar musical intervention *versus* nonfamiliar musical intervention on several aspects of SC in AD patients. We hypothesized that AD patients who listen to familiar songs will have more enhanced aspects of self-consciousness than AD patients who do not listen to familiar songs.

## 2. Methods 

### 2.1. Subjects

 Forty patients with a clinical diagnosis of probable Alzheimer's disease were recruited from 73 AD patients at the Memory Clinic at Salamanca, Spain between 2009–2011. Of the 73 participants recruited, 57 decided to participate in the intervention programs and of these 57, 17 did not attend more than 75% of sessions, which led to their elimination from the analyses. The number of those finally participating in the study was 40. 

All participants were native Spanish speakers and they lived at home with a family caregiver in the provinces of Salamanca, Cáceres, Valladolid and Zamora; most caregivers were the husbands or children of the AD patients and lived with them. All patients were diagnosed at the Department of Neurology of the Hospitals in Salamanca, Valladolid or Zamora.

All AD patients had a history of progressive decline in intellectual function without focal motor or sensory features. To exclude other possible causes of dementia, appropriate laboratory tests were performed and these gave normal results. No findings incompatible with a diagnosis of AD were found in the electroencephalogram, electrocardiogram or chest X-ray in any of the patients. Brain CT scan revealed mild cortical and central atrophy, but no other pathology. All Alzheimer patients met the diagnostic criteria of the “NINCDS—ADRDA Work Group” [42] and of the DSM III-R [43]. All had a score of less than 5 on the Hachinski Ischemic Scale [44]. According to the clinical dementia rating system [35] and mini-mental state—MMS—[45], the patients had mild-moderate AD. Only the AD patients whose verbal comprehension (assessed by the MMS three-stage command) was equal to or above 2 were included in the study. None of the patients had previous musical education, standard musical experiences such as participation in choirs or dance groups, or hearing problems. All AD subjects were taking antidementia medication during the study, in particular antiacetyl cholinesterase treatments.

The patients were divided into two matched groups, according to age, gender, and educational level. Then, one AD patient group was chosen as the experimental AD group and the other as the control AD group, at random. 

Concerning the experimental AD group (18 women and 2 men), the mean duration of the disease was 3.1 years. The AD group had a mean MMS score (±SD) of 19.30 (±3.68) and a mean age (±SD) of 74.38 (±3.56) years. The AD patients had a mean educational level (±SD) of 3.32 (±0.41) using the classification of Barbizet and Duizabo [46]: illiterate (educational level—EL 1—); able to read, to write and to count (EL 2); 6 years of education (EL 3); 9 years of education (EL 4); 11 or 12 years of education (EL 5); 13 years of education (EL 6); and more than 13 years of education (EL 7). 

Concerning the control AD group (19 women and 1 man), the mean duration of the disease was 3.2 years. The AD group had a mean MMS score (±SD) of 19.90 (±2.93) and a mean age (±SD) of 75.15 (±4.23) years. The AD patients had a mean educational level (±SD) of 3.25 (±0.6) according to the classification of Barbizet and Duizabo [46]. 

 The demographic data of the two participating groups are shown in Table 1.

### 2.2. Material

 In the recruitment phase, the patients in the experimental group were asked to state their favourite Spanish music, in particular one familiar song which was well recognised by them. In the case of not naming it spontaneously, we proposed popular songs gathered from the online Spanish traditional music databases (http://tuna.upv.es/asp/liscancion.idc), (http://www.masqueletras.com/buscar.php) confirmed to be wholly familiar to the caregiver and patient in a prescreening survey. In this manner, a list of 11 different familiar songs was chosen for the experimental group. On the other hand, we selected another list of 5 non-Spanish familiar songs for the control group from the same music databases, also confirmed to be wholly nonfamiliar to the caregiver and patient in a prescreening survey. The list of familiar songs was equated with the list of nonfamiliar songs in terms of duration of the song, pleasantness, valence, musical styles, and rhythms.

 Finally, a sung version of each song was created with Apple's Logic Pro 8, after which each one was recorded in technically so that the patients could listen to it at home. Each song had a duration ranging between 1 minute, 46 seconds and 1 minute, 59 seconds. Audio recordings were presented via over-ear ATH-M30 professional headphones from Audio-Technica (http://www.audio-technica.com/) and in the absence of an objective measure of auditory acuity, participants were asked during a pre-intervention example to adjust the volume to a comfortable and clearly audible level. In stimulus presentation the minimum selected headphone volume proved to be between 65 and 70 decibels (dB) and the maximum between 80 and 85 dB.

Additionally, we included a cognitive evaluation using the following general cognitive tests: mini-mental state evaluation—MMSE—[45] and frontal assessment short test—FAS—[47]. 

Self-consciousness was evaluated by the self-consciousness questionnaire [39], which comprises fourteen items, in order to obtain information from the patient about the different aspects of SC (see the questionnaire). Four of these items concerned personal identity (numbers 1, 5, 6 and 7), three of them knowledge of cognitive disturbances (numbers 2, 3 and 4), one self-evaluation of the affective state (number 8), two of them knowledge about representation of the body (numbers 9 and 10), one about anticipation or prospective memory (number 11), one of the capacity for introspection (number 12) and two of moral judgements (numbers 13 and 14). These were simple questions aimed at studying SC as ecologically as possible and in a manner as close as possible to the everyday life of the subjects. The examiners ensured that the answers were understood by the patients for their inclusion in the study. The criteria for rating were carefully determined by two of us as relevant (two points), incorrect (no points), or partly correct (one point). The score obtained for each of these aspects of consciousness was divided by the number of questions corresponding to each aspect. The total maximum score for the seven aspects of SC was 14 points. To contrast the total integrity or alteration of SC, the partly correct and incorrect answers were grouped together as poor answers with respect to “relevant” ones (as good answers). The families helped us to check the accuracy of the answers regarding autobiographical data and the patient's affective state.

### 2.3. Procedure

Pre-intervention assessment was carried out along a 1-2 week period prior to the commencement of the music stimulation and post-intervention assessment, within 1-2 weeks after completion of the music stimulation. All participants were tested individually in a single session. All assessments took between 45–60 minutes to complete. All AD patients underwent two cognitive tests and took the self-consciousness questionnaire [39]. 

In the intervention phase, music stimulation was followed at home by the patient, who was helped by his/her familiar caregiver in the morning. The caregiver received the information and training necessary to carry out music program in one session with us before the intervention phase. The study did not interfere in any way with the patients' pharmacological treatment, nursing care, or in daily activities. None of the patients were participating in any another kind of stimulation program or care. The music stimulation program was carried out in three sessions per week and the participants spent approximately 2–4 minutes at each session. The period of implementation of the music program was approximately 3 months, or 12 weeks, or 36 sessions. On average, the participants who completed the whole intervention period only missed 4% of the interventions. The minimum attendance for not being excluded from the study was 75%. The two patient groups were exposed to the same length of intervention.

The AD patients of the experimental group received a music program consisting of listening to a familiar song, in a passive (no singing along/no movements) but attentive manner. They were also asked to listen to the song in the same quiet room each session, away from potential distracters around from music stimulation, such as a switched-on television, radio, people around them, and so forth. By contrast, the AD patients of the control group received the same music stimulation program, except the song was not familiar. 

### 2.4. Statistical Analyses

 Data were analysed using the Statistical Package for Social Sciences—SPSS—software (version 15.0). Relevant/good and poor answers (incorrect plus partly correct answers) were calculated for each aspect of SC. We defined a treatment benefit as a stabilization or an improvement in the respective outcome measure. To determine the impact of music stimulations on SC, we analysed pre/post-intervention changes in each AD group. To compare the mean scores of SC aspects, Student's *t*-test was used. We also used Pearson's correlations analysis to analyse the relationships between the SC scores and clinical aspects. In this study an *α* level of 0.05 was selected for statistical significance.

## 3. Results

### 3.1. Demographic Characteristics

The number of participants with AD who finally participated in the study was 40. The means and standard deviations of the various demographic characteristics of the control and experimental AD groups are shown in Table 1. 

There were no significant differences in age, gender, or educational level distributions among the groups. In regard to clinical and cognitive aspects, the groups did not differ significantly in the duration of their illness, in the general cognitive state assessed by MMS, or in the frontal functions assessed by FAS.

### 3.2. The Self-Consciousness Questionnaire

 The purpose of our analyses was to evaluate the difference in SC performance between the familiar and unfamiliar song conditions for patients with AD.

At the baseline, there was no significant difference in the SC scores between the AD patients of the control and experimental groups (Table 1). The total score for the SC questionnaire of the experimental AD group was similar to that of the control AD group (mean ± standard deviations: 11.79 ± 2.44 versus 11.98 ± 2.1; *t* = −0.74; and *P* > 0.09). In Table 2 it may be seen that no significant correlation was found between the score on the SC questionnaire and age or educational level in the experimental AD group (*r* = −0.18; *P* > 0.05; *r* = −0.21; and *P* > 0.05, resp.) and in the control AD group (*r* = −0.19; *P* > 0.05; *r* = −0.21; and *P* < 0.05, resp.). However, we did find a significant correlation between the SC score and the duration of AD in both AD groups (*r* = −0.36; *P* < 0.05; *r* = −0.37; and *P* < 0.05, resp.), the SC score and cognitive state evaluated by MMS in both AD groups (*r* = 0.52; *P* < 0.05; *r* = 0.54; *P* > 0.05, resp.) and as well as a significant correlation between the SC score and frontal score evaluated by the FAS test (*r* = 0.48; *P* < 0.05; *r* = 0.45; and *P* < 0.05, resp.) There were no significant differences for any aspect of SC between the experimental and control AD groups (*P* > 0.05). The means and standard deviations of good answers as well as the error percentages for each SC aspect obtained for both groups are presented in Table 3. We also observed that both AD groups showed a similar profile of SC. The experimental and control groups showed consciousness of cognitive disorders (23.8% of wrong answers and 23.3%, resp.) and moral judgements (13.6% and 13.75, resp.) and prospective memory (11.9% and 12.81%, resp.) as the most disturbed aspects of SC, whereas the two least disturbed aspects of SC in both AD groups were personal identity (5.1% and 5.3%, resp.) and representation of the body (3.4% and 3.07%, resp.). 

After the music interventions, we found a slight difference in the total score of the SC questionnaire for the participants in the familiar music stimulation (experimental group), although this difference did not reach the threshold of statistical significance (baseline mean ± standard deviations (SD): 10.11 ± 2.44 versus after familiar music intervention: 11.21 ± 3.14; *P* = 0.056). By contrast, the control group showed a significant increase in impairment for the total SC score (baseline mean ± SD: 10.17 ± 2.1 versus after unfamiliar music intervention: 8.07 ± 1.8; *P* = 0.038). Comparisons between the experimental and control AD groups indicated that there was a significant pre/post-intervention difference in the total scores of the SC questionnaire (*P* = 0.016); (Table 4).

On considering each aspect of SC, the experimental AD group improved significantly in the following aspects of SC after familiar music stimulation: personal identity (baseline mean ± SD: 3.08 ± 1.73 versus after familiar music intervention: 3.48 ± 2.00; *P* = 0.023), affective state (baseline mean ± SD: 0.8 ± 0.07 versus after familiar music intervention: 0.93 ± 0.56; *P* = 0.031), Moral judgements (baseline mean ± SD: 0.9 ± 0.42 versus after familiar music intervention: 1.3 ± 2.21; *P* = 0.037), and body representation (baseline mean ± SD: 1.9 ± 0.23 versus after familiar music intervention: 1.99 ± 0.17; *P* = 0.049). Anosognosia (baseline mean ± SD: 2.03 ± 2.31 versus after familiar music intervention: 2.14 ± 1.96; *P* > 0.05), Prospective memory, (baseline mean ± SD: 0.65 ± 2.1 versus after familiar music intervention: 0.63 ± 2.4; *P* > 0.05) and Introspection (baseline mean ± SD: 0.75 ± 1.54 versus after familiar music intervention: 0.74 ± 1.65; *P* > 0.05) remained unchanged after familiar music intervention. In contrast, when the control AD group received the unfamiliar music program, impairment in most aspects of SC (*P* < 0.05) increased, except Body Representation, which improved significantly 1.72 ± 0.32 (mean ± SD) to 1.88 ± 1.54 (*P* = 0.049), and Affective State, which remained unchanged (baseline mean ± SD: 0.79 ± 0.1 versus after familiar music intervention: 0.77 ± 0.41; *P* > 0.05).

Comparisons between the experimental and control AD groups indicated that there were significant pre/post-intervention differences in the following SC aspects between both AD groups: Personal identity (*P* = 0.019), Affective state (*P* = 0.02), moral judgements (*P* = 0.034), prospective memory (*P* = 0.04), and anosognosia (*P* = 0.042). We did not find significant pre/post-intervention differences in the following SC aspects between both AD groups: Body representation (*P* > 0.05) and introspection (*P* > 0.05). By contrast, there were significant pre/post-intervention changes in the scores on the MMS and FAS tests (*P* < 0.05) in the control AD group, but not in the experimental AD group. The control AD group showed poorer scores in the post-intervention phase (MMS: mean ± SD at baseline: 19.90 ± 2.93 versus after the intervention phase: 17.35 ± 3.57; FAS: 9.11 ± 2.15 versus 6.93 ± 3.41), whereas the experimental AD group did not vary in their cognitive performance (MMS: mean ± SD at baseline: 19.30 ± 3.68 versus after the intervention phase: 19.5 ± 3.28; FAS: 9.33 ± 2.37 versus 9.04 ± 3.14), (Table 5). 

## 4. Discussion 

The first important aspect is that we observed the same SC profiles between both AD groups. In addition, we found similar SC profiles between our AD patients and those in the study conducted by Gil et al. [39], who also used the SC questionnaire. AD clearly induced a heterogeneous impairment of SC but a similar degree of impairment in all participants in both studies. The most impaired SC aspects in both studies were, in descending order: anosognosia, moral judgement and prospective memory, whereas the best preserved aspects of SC in all AD patients were Personal Identity and body representation. Accordingly, to our knowledge we had replicated the results of the only study on SC in mild-moderate AD using the SC questionnaire.

The main goal of the present study was to determine the impact of familiar music on self-consciousness in AD patients. Considering the beneficial effect of music on SC, such as stabilization or an improvement in the SC score, the results confirmed our hypothesis that patients with AD who listened to familiar songs had an enhanced self-consciousness. However, the control AD group who received unfamiliar music stimulation did not show any stabilization or improvement of the SC score after the music intervention. This result could be explained in terms of a link between SC and cognition [39, 48]. We observed a general cognitive deterioration, reflected by poorer scores on the MMS and FAS tests in the AD patients of the control group in the post-intervention phase, whereas the experimental AD group maintained similar MMS and FAS scores. We also observed relationships between the SC scores and the scores on the MMS and FAS tests. Nevertheless, our results did not permit us to distinguish whether this cognitive deterioration in the control group was due to the course of the disease or to the music intervention, to both, or even to other aspects, such as the treatments implemented. Regarding the general results, familiar music could be considered as an enhancer not only of SC in AD, but also of the general cognitive state [49]. Another possible explanation for the observed dissociation in favour of familiar songs on SC is that unfamiliar songs could depend more on episodic memory than familiar songs. In AD patients, where general cortical and hippocampal atrophy impairs standard episodic learning, familiar music stimuli can allow a more diversified cognitive functioning. In addition, Gil et al. [39] suggested that SC requires a convergence of many neural networks and that the core deficiency in AD patients might be impaired SC, equated with the disability to maintain sequential and simultaneous “attention to life” [50, 51]. The music processing also encompasses a complex neural network that recruits information from all areas of the brain, including suborbital areas such as the basal ganglia, nucleus accumbens, ventral tegmental area, hypothalamus, and cerebellum [23, 52–56], and cortical areas such as the medial prefrontal cortex [57] and orbitofrontal cortex [56], which are affected more slowly in AD compared to the areas of the brain typically associated with memory [58]. Thus, familiar music may create a more robust SC in patients with AD. 

The impact of music stimulation on SC was heterogeneous. In the experimental AD group, the scores on aspects of anosognosia, prospective memory and introspection were maintained after the intervention phase, and we found—in descending order—an improvement in the aspects of personal identity, affective state, moral judgements and body representation. In contrast, the control AD group showed a deterioration most of the SC aspects after unfamiliar music stimulation, except the SC aspects of body representation and Affective State. The most deteriorated aspects of SC in the control group were anosognosia and Prospective memory. Anosognosia, prospective memory, and introspection are usually more dependent on frontal functioning [59–62] than another cognitive aspects such as autobiographical memory or representation of the body. In general, it could be that the impact of music is lower on cognitive aspects that are more related with frontal functioning. The experimental AD group increased the SC aspects of autobiographical memory and mood after familiar song intervention. There is evidence pointing to the positive effect of music on autobiographical data in AD patients [20–22]. The study of Irish et al. [21] noted the enhancing effect of music on autobiographical memory recall in mild Alzheimer's disease individuals, using one music condition and another one in the silence condition. They also observed a significant reduction in the anxiety status of AD patients in the music condition, suggesting a reduction in anxiety as a potential mechanism underlying the enhancing effect of music on autobiographical memory recall. Nevertheless, this study used Vivaldi's “Spring” movement from “The Four Seasons”, which is a famous music score, for the music condition but the authors did not explain whether this music was familiar or unfamiliar to all participants. However, El Haj et al. [20] found an enhancement of autobiographical recall of AD patients in the familiar music exposure condition versus the silence condition. They observed that compared with memories evoked in silence, the memories evoked in the music condition were accompanied by more emotional content and impact on mood, were retrieved faster and engaged less executive processes. These observations could support the notion that music-evoked autobiographical memories contain all the features of involuntary memories. Conway and Pleydell-Pearce [40] offer a theoretical explanation of about the retrieval of involuntary music-evoked memories, which emphasize the importance of perceptual cues in triggering associative retrieval. In our produce, familiar music has all the features of a perceptual cue, because it is freely chosen by experimental group, is also likely to be closely associated with highly active and accessible personal goals and thus, activates associative retrieval. Following this line of thought, we suggest that familiar songs should cue involuntary memories by favouring events formerly associated with that cue.

Halpern and O'Connor [10] suggested the participation of implicit memory for musical stimuli to remain intact, it could be that implicit memory for familiar songs could help the SC of emotional life events to emerge [11]. Regarding neural basis, SC, emotion, and implicit memory share neural subcortical structures such as the amygdales or basal ganglia, which are ontogenetically older than those of explicit memories. Familiar music could induce oscillatory synchrony in neural loops associated with implicit memory or verbal memory [63] and such synchronous neuronal firing may support this more composite SC process. Furthermore, music and dancing skills learned over the years often occur automatically well into the later stage of dementia, due to implicit/procedural processes, and this could allow the SC of body representation to emerge. It could allow AD patients who have lost other social and cognitive skills to participate and gain a sense of their own existence.

 Additionally, the affective nature of the familiar songs in the present experiment deserves further consideration. All songs in our study were popular or children's songs and this might have enhanced concentration during the intervention phase, resulting in more focused “attention to life” or self-consciousness. However, the SC of affective State was stabilized after unfamiliar music intervention, which could be explained in terms of similar, simple, and repetitive melodic structures of unfamiliar and familiar popular/children's songs. In this sense, Hill and Palmer [34] observed that new musical stimuli can elicit affective responses. There is also evidence concerning the positive effect of familiar music on affective state [20, 24, 30–33] and in general, on depression and anxiety [64, 65]. 

Our study also found that the representation of the body improved after unfamiliar and familiar music interventions. This result could be explained in terms of the information given by the caregivers of both groups, who informed us that their relatives with AD produced many body movements when listening to music. In general, the feedback from the relatives, in an informal and spontaneous way, regarding the results of the study was positive because music activity helped them to be closer to the patients, although the length of the proposed sessions was an area identified as requiring further consideration. However, the caregivers of the AD patients that received familiar music stimulation informed us more frequently than those of the control AD group, and the former patients showed many emotional reactions, such as singing, motor movements, interactions with caregivers, and spontaneous language. This could be also due to the familiarity of songs generating inputs that led them to be more conscious of their bodies. Also, it is possible that the group given familiar music were motivated to sing spontaneously outside the intervention session or to listen more to music, thus, enhancing the size of the effects, but we have not been able to record those data from caregivers. Studies such as those of Clair and Bernstein [24] or Götell et al. [30] have suggested that music has an exceptional ability to elicit memories, movements, motivations, and emotions from older adults affected by dementia because of the familiarity and predictability of music. Thus, music programs, independently of familiarity with the music, could enhance self-consciousness of moods and body representation in AD patients.

 To conclude, the present study permitted us to observe an enhancement, through familiar music stimulation, of self-consciousness in AD patients. This impact is heterogeneous in the different aspects of SC. It would be recommendable to program familiar music interventions at home as well as in geriatric institutions in order to allow patients to express feelings, to facilitate meaningful interactions and to elicit positive reactions (e.g., eye contact, meaningful vocalization), leading to a reduction in agitation, depression, and restlessness. These types of music interventions also provide an opportunity for family members to reestablish emotional closeness and meaningful interactions that may have been lost due to the AD, appreciably enhancing the quality of life of both the patients and their family. It is possible that music stimulation delivered in group settings could permit patients to receive cues to increase their social interactions [66], which have been linked to the maintenance of cognitive and emotional functioning [67] and social skills [68]. It would be interesting to perform future studies in order to understand the nature of familiar musical processing in patients with AD, which could allow the development of effective and comprehensive musical interventions for this disease. In legal and clinical practice, the power of familiar music as an SC enhancer should be considered more often when assessing the cognition performance of AD patients or making decisions about the future course of personal events.

## Figures and Tables

**Table 1:** Means (M) and standard deviations (SD) of several demographic, and clinical characteristics of the experimental and control AD groups.

	AD experimental group	AD control group	*P* value^a^
	M (SD)	M (SD)	
Sample size (*N*)	20	20	
Gender (F : M)	18 : 2	19 : 1	>.05
Age (years)	74.38 (3.56)	75.15 (4.23)	>.05
Educational level	3.32 (0.41)	3.25 (0.6)	>.05
Duration of illness (years)	3.1 (1.8)	3.2 (1.3)	>.05
Clinical dementia rating (CDR)	1.2 (0.3)	1.3 (0.5)	>.05
MMS	19.30 (3.68)	19.90 (2.93)	>.05
FAS	9.33 (2.37)	9.11 (2.15)	>.05
Self-consciousness questionnaire	10.11 (2.44)	10.17 (2.1)	>.05

^a^ *P* values referred to control and experimental groups.

*Significant difference (*P* < .05).

**Table 2:** Correlation coefficients between the self-consciousness test score and the demographic, and clinical characteristics of the experimental and control AD groups.

	AD experimental group	AD control group
Age (years)	−0.18	−0.19
Educational level	−0.21	−0.21
Duration of illness (years)	−0.36*	−0.37*
MMS	0.52*	0.54*
FAS	0.48*	0.45*

*Significant difference (*P* < .05).

**Table 3:** Error percentages and means and standard deviations (SD) of good answers for each aspect (A) of self-consciousness produced by the experimental and control AD groups, before the intervention phase (at baseline).

Aspects of self-consciousness questionnaire	Experimental AD group		Control AD group		*t*	*P* ^a^
	Mean (SD) %	Error	Mean (SD)	Error %		
A1: personal identity	3.08 ( 1.73)	5.1	3.26 ( 1.48)	5.3	−1.41	NS
A2: anosognosia	2.03 (2.31)	23.8	1.99 (2.44)	23.3	0.86	NS
A3: affective state	0.8 (0.07)	6.8	0.79 (0.1)	6.71	0.26	NS
A4: body representation	1.9 (0.23)	3.4	1.72 (0.32)	3.07	1.03	NS
A5: prospective memory	0.65 (2.1)	11.9	0.7 (1.91)	12.81	−0.19	NS
A6: introspection	0.75 (1.54)	8.5	0.8 (1.63)	9.06	−0.24	NS
A7: moral judgments	0.9 (0.42)	13.6	0.91 (0.61)	13.75	−0.12	NS

NS: not significant (*P* > .05).

Error %: percentage of poor answers (incorrect plus partly correct answers).

^a^ *P* values referred to the means of the control and experimental groups.

**Table 4:** Means and standard deviations (SD) of good answers for each aspect (A) of self-consciousness test produced by the experimental and control AD groups in the pre- and post-intervention phases.

Aspects of self-consciousness (SC) questionnaire	Experimental AD group		*t*	*P*	Control AD group		*t*	*P*	*P* ^a^
	Mean (SD)	Mean (SD)			Mean (SD)	Mean (SD)			
	Pre-	Post-			Pre-	Post-			
A1: personal identity	3.08 ( 1.73)	3.48 (2.00)	−2.63	.023	3.26 ( 1.48)	2.84 ( 1.42)	2.21	.044	.019
A2: anosognosia	2.03 (2.31)	2.14 (1.96)	−0.67	NS	1.99 (2.44)	1.03 (2.63)	2.46	.027	.042
A3: affective state	0.8 (0.07)	0.93 (0.56)	−2.34	.031	0.79 (0.1)	0.77 (0.41)	0.11	NS	.02
A4: body' representation	1.9 (0.23)	1.99 (0.17)	−2.19	.049	1.72 (0.32)	1.88 (1.54)	−2.29	.049	NS
A5: prospective memory	0.65 (2.1)	0.63 (2.4)	0.23	NS	0.7 (1.91)	0.3 (3.1)	2.71	.019	.04
A6: introspection	0.75 (1.54)	0.74 (1.65)	0.22	NS	0.8 (1.63)	0.61 (2.34)	2.27	.048	NS
A7: moral judgments	0.9 (0.42)	1.3 (2.21)	−2.38	.037	0.91 (0.61)	0.64 (0.98)	2.36	.045	.034
Total score of SC	10.11 (2.44)	11.21 (3.14)	−2.07	.056	10.17 (2.1)	8.07 (1.8)	2.44	.038	.016

^a^ *P* values referred to comparison of pre/post-intervention differences between the experimental and control AD groups.

**Table 5:** Means and standard deviations (SD) of MMS and FAS tests produced by the experimental and control AD groups in the pre- and post-intervention phases.

	Experimental AD group		*P*	Control AD group		*P*
	Mean (SD)	Mean (SD)		Mean (SD)	Mean(SD)	
	Pre-	Post-		Pre-	Post-	
MMS	19.30 ( 3.68)	19.5 (3.28)	NS	19.90 ( 2.93)	17.35 (3.57)	.038*
FAS	9.33 (2.37)	9.04 (3.14)	NS	9.11 (2.15)	6.93 (3.41)	.009*

*Significant difference.

NS: not significant.
